# The Book World of Medicine and Science

**Published:** 1903-10-10

**Authors:** 


### The Book World of Medicine and Science.

Philips' Anatomical Model of The Female Body. Edited by W. S. Furneaux. (London: George Philip and Son, Limited. 1903. Price 4s. net.)

This coloured model is an attempt to give a pictorial representation of the human body and its organs, and as such we believe it will prove serviceable to medical students who are just commencing their anatomical studies, and more especially to nurses, who do not as a rule have the opportunity of learning from actual dissections. Good models are very useful things for teaching purposes, and the futility of attempting to convey concepts of anatomical structures in the absence of either models or dissections, must be pretty clear to everyone who has made the attempt, be he never so adept with chalk and blackboard. Accompanying the model under consideration are 16 pages of simple and well-chosen letterpress. We have one fault to find with the coloured plates, and that is their want of accuracy in detail. We do not think it either advisable or necessary to portray the tarsal bones as a confused jumble in which the astragalus and os calcis are fused into one mass; and it must be confessed that were we shown a humerus which resembled the illustration of that bone in Plate E, we should strongly deny it any human kinship. However, it is nearly always easy to find fault with anatomical representations, and, in spite of the slight drawbacks we have mentioned, the model under consideration is a move in the right direction and deserves to have a large sale.

International Clinics. Vol. II., Thirteenth Series. (London: J. B. Lippincott Company. Pp. 311. With Illustrations. 1903. Price 10s. 6d.)

We have previously had occasion to draw attention to the excellence of these "International Clinics," and the volume now under consideration fully maintains the high standard of worth attained by its predecessors. It is not too much to say that every article the book contains is thoroughly deserving of careful study and attention. The subjects which are dealt with are arranged under the following sections: the summer diarrhœas of children, disease of the pancreas, treatment, medicine, surgery, pediatrics, obstetrics and gynæcology, and ophthalmology. Where all is so good it may seem invidious to draw special attention to individual sections without discussing the remainder. Nevertheless, the articles on the summer diarrhœas of children and on disease of the pancreas may be given especial prominence, both on account of the attention which is being given to these subjects at the present time, and because of the intrinsic value of the individual papers, while Professor Edmund Landolt's article on surgical intervention in paralysis of the ocular muscles is deserving of careful scrutiny by everyone who is interested in eye work. He makes a strong argument against the operation of tenotomy of the eye muscles in the treatment of ocular paresis or paralysis, and compares the man who would perform the operation to a surgeon who, in order to remedy a paralysis of the extensor muscles of the forearm, would cut the tendons of the flexors. The only operation to be done, according to Professor Landolt, when all other treatment has failed, is advancement of the affected muscle with, in some instances, resection of its tendinous attachment. Although this method does not fully accord with our notions of restoring activity to a weakened muscle by preventing it from being overstretched by the opposing muscles, and in spite of the fact that other operators have expressed dissatisfaction with the method, Professor Landolt speaks with conviction of the success which has followed in his own cases, and any opinion coupled with his name will of course carry great weight.

Essays on Rural Hygiene. By George Vivian Poore, M.D., F.R.C.P., Emeritus Professor of Medicine and Clinical Medicine, University College, London, etc. (Pp. 426. With 12 illustrations. Third edition. London: Longmans, Green and Co. 1903. Price 6s. 6d.)

Dr. Vivian Poore's charming collection of studies on rural sanitation first appeared in the spring of 1893, and a second edition followed in the autumn of 1894. The third edition issued this summer would seem to indicate that the common-sense views expressed and the rational methods enthusiastically advocated were at last gaining the attention they deserve. The author is no mere fashionable sanitarian blindly following custom and willingly walking in the steps of present-day procedure. He is fully convinced that our elaborate and expensive methods of sanitation which seem to most of us necessary under conditions of home life are unsuited to the needs of the rural householder who is dependent for his living upon the productions of the soil, and who should be taught to return all refuse matter to the soil with a view to increase its fertility. Dr. Poore is an original thinker, a practical worker, a critic who bases his views on the sound rock of experiment, and urges his opinions from the rational platform of experience. The shortcomings of modern sanitation and the follies of many would-be sanitarians are freely revealed in vigorous English. It is urged that the mixing of putrescible matter with water is a fundamental scientific error which leads to the dissemination of water-borne diseases, the pollution of rivers and the poisoning of wells. The chapter dealing with "The Living Earth" is peculiarly rich in valuable suggestions. It is shown that the humus possesses a marvellous power not only of turning organic matter into food for plants by what is known as the process of nitrification, but that while in this way it tends to increase our food supplies, it is no less powerful, if rightly and scientifically used, to protect our wells from all dangerous animal pollutions. Dr. Poore's views on what he terms the "earth-unit" merit serious consideration, and if sometimes an enthusiasm for natural methods seems to lead him to neglect the peculiar difficulties of adapting such to modern manners of life, his style of presentation is so rich in attractiveness, and the method of expression so plentiful in sweet reasonableness, that criticism is in great measure disarmed. The chapters dealing with the author's personal experiences in attempting to solve the problem of domestic sanitation form the most fascinating sections of this peculiarly suggestive work. In a chapter on "Burial," Dr. Poore contends that earth burial is not only safe, but that its safety lies in absolute simplicity, and he holds that cremation of a body involves a needless dissipation of energy. The concluding section contains an interesting account of the reclamation of the sand wastes of Gascony. A reviewer may well consider it a task of supererogation to praise a third-edition work, but we should like to add that Dr. Poore's work is of such a character that it may well be studied with care by every medical man, intelligent nurse, and thoughtful layman desirous of making the most of this country, and bringing the greatest good to the greatest number of its people.

